# Innovation in Cassava Bagasse Valorization: Efficiency of Convective Drying Enhanced with Ultrasound and Pulsed Electric Fields

**DOI:** 10.3390/foods13172796

**Published:** 2024-09-02

**Authors:** José G. Serpa-Fajardo, Elvis J. Hernández-Ramos, Ricardo D. Andrade-Pizarro, Alberto A. Aguilar-Lasserre, Gregorio Fernández-Lambert

**Affiliations:** 1 Postgraduate Studies and Research Division, Tecnológico Nacional de México/ITS de Misantla, Misantla 93850, Veracruz, Mexico; jose.serpa@unisucre.edu.co (J.G.S.-F.); elvis.hernandez@unisucre.edu.co (E.J.H.-R.); 2 Faculty of Engineering, Universidad de Sucre, Sincelejo 700001, Colombia; 3 Department Food Engineering, Universidad de Córdoba, Montería 230002, Colombia; rdandrade@correo.unicordoba.edu.co; 4 Division of Research and Postgraduate Studies, Tecnológico Nacional de México/Instituto Tecnológico de Orizaba, Veracruz 94320, Mexico; albertoaal@hotmail.com

**Keywords:** emerging technologies, combined techniques, agro–industry, starch, sustainability

## Abstract

This research proposes an efficient alternative for dehydrating cassava bagasse to address the inherent challenges in the handling, transportation, storage, and preservation of this agro–industrial residue generated in cassava starch production plants. This residue is characterized by high moisture retention, considerable volume, and hydrophilic nature, complicating conventional drying methods. This study evaluates the impact of emerging ultrasound (US) and pulsed electric field (PEF) technologies prior to convective drying to enhance the dehydration efficiency of cassava bagasse, aiming at its valorization and contributing to the sustainability of the cassava starch industry. The findings reveal that pretreatment with ultrasound (US) and pulsed electric fields (PEF) significantly reduces the drying time of cassava bagasse compared to convective drying alone. With probe ultrasound at 26 kHz for 30 min, the drying time is reduced by 72% (3.83 h vs. 14.0 h); with bath ultrasound at 37 kHz for 30 min, it is reduced by 56.0% (6.16 h vs. 14.0 h); and with PEF at 7.5 kV/cm for 30 min, it is reduced by 52.4% (6.66 h vs. 14.0 h). These emerging technologies increased the effective diffusivity and modified the molecular structure of the bagasse, thereby improving mass transfer and drying process efficiency. These results are particularly useful for developing more efficient and sustainable strategies for drying agricultural by-products, with direct implications for the post-industrial treatment of agro–industrial residues with high water content.

## 1. Introduction

The removal, control, and management of moisture in agro–industrial waste are important, both for reasons of negative externalities and for their value in post-industrial management. In this regard, improving processing efficiency through comprehensive resource utilization and reducing energy consumption and operating costs in an environmentally friendly manner are always goals of any industry, particularly the agri–food industry [1].

To decrease the moisture content of raw materials or by-products from the food industry, drying is the most used operation. However, in hydrophilic and high-moisture products, it has the disadvantage of requiring long operation times with high energy consumption, and consequently, high operating costs. As a response to this issue, emerging technologies, combined with traditional thermal processes, have been proposed to improve convective drying efficiency [1,2,3].

Among the emerging technologies applied for this purpose, ultrasound (US) and pulsed electric field (PEF) technologies stand out due to their simplicity and cost-effectiveness. They have been successfully used to improve the drying efficiency of various agri–food products with lower water retention than cassava bagasse residue, such as fruits and vegetables, tubers like potatoes, cassava, yams, and sweet potatoes [3,4,5], and even in some agro–industrial residues like mango peels [6], orange peels [7,8], and pomegranate peels [9].

The frequencies of sound waves generated by US produce acoustic cavitation, causing expansion, contraction, rupture, and a series of physical disturbances in the material’s structure. Additionally, it generates alternating positive and negative pressure that pressurizes and relaxes the materials, along with the effect of creating micro-channels in the cellular structure, promoting rapid and efficient mass transfer, allowing moisture to move more freely and accelerating the drying process of the products [4]. Similarly, the application of PEF induces fracture and pore formation in cell membranes through ion polarization, facilitating the permeability of the cell membrane. This disintegration of the natural structure of the cell membrane contributes to water loss processes during osmotic dehydration and subsequent drying of the products due to increased mass and heat transfer, resulting in greater efficiency in the process [10,11].

The application of US and PEF as pretreatments to convective drying has reduced drying time by between 50 and 70%, depending on the structure, hardness, and porosity of the product [10,11,12].

To address and scale US-assisted drying processes, establishing drying kinetics is fundamental [4]. In this context, to model the material transfer related to water loss during cassava bagasse drying using US and PEF pretreatments, diffusion models based on Fick’s second law, as well as other empirical models, can be considered. Although these empirical models do not explicitly incorporate factors or parameters of emerging technologies, their effects on the process can be evaluated by considering their influence on drying kinetics [4].

Although emerging technologies of US and PEF, when applied as pretreatments to convective drying, have demonstrated improved process efficiency, their effects on drying hydrophilic agro–industrial residues with high moisture content have been little studied. An example of such agro–industrial residues is cassava bagasse generated in the cassava starch industry, which is currently utilized for animal feed [13]. However, although there are studies for its utilization in the biotechnological field [14] and in biomaterials [15], the problem of its handling, storage, and transportation remains an environmental and economic challenge for post-industrial utilization due to its high water content, which is approximately 85–90% [16].

This problem worsens environmentally due to the high volumes of this agro–industrial waste generated, often deposited in open areas, where all kinds of microorganisms proliferate, leading to uncontrolled fermentation and foul odors that affect both production plants and surrounding communities.

This research evaluates the effects of US and PEF technologies as pretreatments to convective drying of cassava bagasse. The combination of traditional techniques, such as convective drying, with these emerging technologies, represents an innovative approach to enhancing drying efficiency and the post-industrial value of agro–industrial waste. This study significantly contributes to advancing knowledge in the field of drying agri–food products and opens new research avenues for developing comprehensive strategies for utilizing waste with high moisture content. By addressing the challenges of handling, transportation, storage, and preservation of these wastes, a more sustainable and energy-efficient industry is promoted in environmental and economic terms.

## 2. Materials and Methods

### 2.1. Sample Collection

The sample was provided by a cassava starch producer (Almidones de Sucre S.A.S., Morroa, Colombia), taken from the stacking of cassava bagasse discharged from the starch extraction process. An approximate sample of 3 kg was divided into roughly three equal portions and placed in separate percolators for 2.5 h to reduce the water content accompanying it from the extraction process. Subsequently, the cassava bagasse was combined and homogenized again.

### 2.2. Proximate Characterization of Cassava Bagasse

Cassava bagasse was analyzed for moisture content, ash, fat, fiber, and protein content based on the following standards [17]: 977.11 AOAC oven method; 942.05 AOAC general method; 920.39 AOAC Soxhlet method; 962.09 AOAC gravimetric chemical method, and 955.04 AOAC Kjeldahl method, respectively. Total carbohydrate content was determined by difference using Equation (1).

(1) $$\%CT=100-\left(\%H+\%G+\%Ce+\%F+\%Pc\right)$$

where CT is total carbohydrate; H is moisture; G is fat; Ce is ash; F is crude fiber; Pc is protein.

### 2.3. Ultrasound Application

With the purpose of determining the impact of the type of ultrasound equipment on the efficiency of the cassava bagasse drying process, the application of US was carried out as a pretreatment to convective drying using two types of equipment: a bath-type US Elmasonic P 60 H (Made in Singen, Germany by Elma) with temperature control at 34 °C and a probe-type US UP200St (Made in Teltow, Germany by Hielscher). The bath-type US was applied with a power of 100 W and an ultrasonic frequency of 37 kHz, while the probe-type US was applied with a power of 77 W and a frequency of 26 kHz using a Sonotrode S26d7D (Made in Teltow, Germany by Hielscher) with a 7 mm diameter. With both pieces of equipment, the sonication process of the cassava bagasse was performed using 300 g of a product sample in a 5% saline solution, with a solution-to-sample ratio of 3.58 g. The application time of US was 10 and 30 min, respectively, with mechanical agitation at 320 rpm.

### 2.4. Application of Pulsed Electric Fields

**Figure 1:**
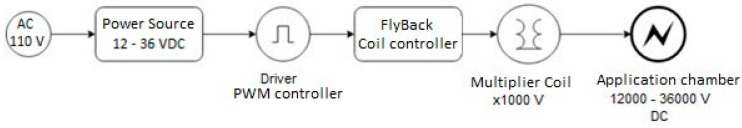
Diagram of the PEF system components.

The PEF was applied as a pretreatment to convective drying using a prototype PEF square wave device as shown in Figure 1.

The PWM (Pulse Width Modulation, Generic brand made in China) controller is an electronic device designed to generate a pulse train by modulating the width, with a frequency of 45 μs. The FlyBack CC12-30V (Generic brand made in China) coil controller was used to generate the appropriate wave patterns by frequency for the Multiplier Coil, responsible for increasing the DC voltage with a multiplication factor of 1000 V. The calibration of the prototype device, in terms of pulse width and field intensity, was performed using a Gw INSTEK GDS-2102A oscilloscope (Digital Storage Oscilloscope, 100 MHz, 2 GS/s, Made in Taipei, Taiwan by Gw Instek).

The electric field intensity (E) in kVcm^−1^ was determined according to Equation (2), where V is the electrode voltage [kV] and d is the distance between two electrodes [cm].

(2) $$E=\frac{V}{d}$$

The application of PEF on cassava bagasse was conducted on a 125 g sample of cassava bagasse in a 5% saline solution, with a solution-to-sample ratio of 3.58 g of solution per gram of sample. Electric field intensities of 2.5 kVcm^−1^ and 7.5 kVcm^−1^ were used, with treatment times of 10 and 30 min.

### 2.5. Drying Operation

After applying the US and PEF processes, the samples were drained for 20 min using a #12 mesh sieve. Subsequently, the samples were placed in the convective drying chamber of a tray dryer model TD-c/EV from Electrónica Véneta S.P.A., Treviso, Italy, with a thickness of 5 mm and a drying area of 0.0225 m^2^, under controlled temperature conditions of 40 ± 1 °C, provided by nine resistors of 300 W each, and an air velocity of 1.5 m/s transmitted by a 300 W fan.

The experiment excluded ambiguous factors such as temperature variation or drying air velocity to accurately evaluate the effects of US and PEF pretreatment on the drying efficiency of cassava bagasse. Keeping these latter conditions constant ensures that the observed effects are due solely to the pretreatment techniques and not to variations in the drying conditions.

#### Drying Kinetics

The weight loss of each sample was determined using a KERN KB^®^ digital electronic balance (Made in Zigelei, Germany by Kern & Sons) with a sensitivity of 0.1 g. Measurements were taken at intervals of 5 min during the first 30 min, every 10 min up to 90 min, every 15 min up to 210 min, and, finally, every 30 min until the sample reached equilibrium moisture under the employed drying conditions.

To evaluate the effects of US and PEF pretreatments on the convective drying process of cassava bagasse, the drying time required to reach equilibrium moisture was determined as the response variable. Additionally, the drying kinetics were assessed by recording the moisture ratio (MR) as a function of time, and nine empirical models (Table 1) were evaluated as referenced by Salcedo et al. [18] to describe the convective drying kinetics of cassava bagasse without pretreatments.

The determination of the best-fitting model to the experimental drying data of cassava bagasse with US and PEF pretreatments was based on the coefficient of determination, R^2^, and the root mean square error (*RMSE*) (Equation (3)). In this equation, *MRmod* represents the moisture ratio predicted by the model, and *MRexp* represents the experimental moisture ratio.

(3) $$RMSE=\frac{1}{N}\sum_{i=1}^{n}\left(MRmod-MRexp\right)$$

The moisture ratio of cassava bagasse was established according to Fick’s second law (Equation (4)). In this equation, *C* represents the moisture concentration, *t* is time, *Dv* is the diffusion coefficient, and *z* is the characteristic dimension.

(4) $$\frac{\partial C}{\partial t}=-Dv\frac{\partial^{2}C}{\partial z^{2}}\;$$

The average diffusion coefficient (*Dv*) was determined by fitting the experimental data to a straight line obtained from the natural logarithm (Ln) of the moisture ratio (*MR*) as a function of drying time in hours and comparing it to the theoretical value of Ln (*MR*) according to Equation (5). In this equation, *Dv* is the diffusivity (m^2^/s), t is the drying time (h), and *z* is the sample thickness (m). The moisture ratio (MR) is calculated according to Equation (6), where *Xt* is the moisture at time *t*, *Xequil* is the equilibrium moisture, *Xt*1 is the initial moisture, *X* is the free moisture at time *t*, *X*1 is the initial free moisture at time *t* = 0, *a*1 = ${\left(\frac{\pi }{2}\right)}^{2}$, and *Fo* is $\frac{\left(Df\;t\right)}{z^{2}}$.

(5) Dv=Dv tz2theor (for a flat sheet)∗ z2ttexperimental

(6) $$MR=\frac{\left(Xt-Xequil\right)}{\left(Xt1-Xequil\right)}=\frac{X}{X1}\;$$

The integration of Equation (4) leads to Equations (7) and (8). However, for long processing times (*Fo* > 0.1) characteristic of diffusion drying phenomena, only the first term of the series is considered, as expressed in Equation (9).

(7) $$MR=\frac{\left(Xt-Xequil\right)}{\left(Xt1-Xequil\right)}=\frac{X}{X1}=\frac{8}{\pi^{2}}\;\sum_{n=1}^{\infty }\frac{1}{{\left(2n-1\right)}^{2}}\;Exp\left[{-(2n-1)}^{2}\frac{Dv\;\pi^{2}t\;}{4z^{2}}\right]$$

(8) $$MR=\frac{8}{\pi^{2}}\;\left[e^{-a1\;Fo}+\frac{1}{9}\;e^{-9a1\;Fo}+\frac{1}{25}\;e^{-25a1\;Fo}+\ldots \right]$$

(9) $$MR=\frac{X}{X1}=\frac{8}{\pi^{2}}\;Exp\left(\frac{-Dv\;\pi^{2}t\;}{4z^{2}}\right)\;$$

### 2.6. Energy Consumption

The energy consumed (*E*, kWh), as an economic indicator, was determined for the three best treatments using US or PEF as pretreatments for drying cassava bagasse and for treatment T0 where no pretreatment is applied. This energy consumption was determined according to Equation (10), where *U* is the voltage (V), *I* is the current intensity in amperes (A), *W* is the electrical power in watts (*W*), *R* is the electrical resistance in ohms (Ω), and *t* is the treatment time.

(10) $$E=\int_{0}^{\infty }\frac{{U(t)}^{2}}{R}dt=\int_{0}^{\infty }U\left(t\right)\;I(t)dt=\int_{0}^{\infty }W\left(t\right)dt$$

The calculations considered the described characteristics of the US, PEF, and drying equipment used, as well as their respective operating times.

### 2.7. Infrared Spectroscopy

An infrared spectrometer was used to evaluate changes in the conformation of functional groups in cassava bagasse for T0 and the best treatments after applying US and PEF before convective drying of cassava bagasse. Infrared spectra were obtained in the region of 500 to 4000 cm^−1^, performing 32 reading scans at a resolution of 8 cm^−1^, using a single bounce ATR accessory (PerkinElmer UATR-L1600107, made in Shelton, CT, USA by PerkinElmer) with a 1.5 mm diameter diamond crystal.

### 2.8. Residual Starch

The residual starch content was determined for the three best treatments using US or PEF as well as for treatment T0 where no pretreatment is applied. It was determined by enzymatic hydrolysis with alpha-amylase, following the procedure reported by Ref. [19] with some minor modifications. An amount of 200 mg of the sample was suspended in 40 mL of distilled water and dextrinized with 20 μL of alpha-amylase at 90 °C for 15 min with constant stirring. To the dextrinized sample, 2.5 mL of 0.1 M sodium acetate-acetic acid buffer (pH 4.8) was added. Subsequently, the temperature was adjusted to 60 °C, and 200 μL of amyloglucosidase was added for 30 min. The glucose obtained was reacted with 3,5-dinitrosalicylic acid (3,5-DNS) to determine the concentration of reducing sugars.

### 2.9. Field Emission Scanning Electron Microscopy (FESEM) Morphological Characterization

The morphological and surface analysis of the dried cassava bagasse was conducted using Field Emission Scanning Electron Microscopy (FESEM, Thermo Fisher Scientific, model Apreo 2 S, made in Adelaide, Australia), following the procedure based on ASTM E1508-12a standard, utilizing a Field Emission Scanning Electron Microscope (FESEM) and an Energy Dispersive Spectroscopy (EDS) analyzer (Thermo Fisher Scientific, model ANAX-30P-B UltraDry 30 mm^2^, made in Adelaide, Australia). This analysis was performed for T0 and the best treatments after applying US and PEF as pre-treatments before convective drying.

### 2.10. Experimental Design

To evaluate the effects of using US and PEF cassava bagasse, a completely randomized design (CRD) with nine treatments was used as described in Table 2. All tests were conducted in triplicate.

The statistical analysis was conducted using R-Studio v.2.9.1 (The R Foundation for Statistical Computing, 2009). An ANOVA was applied with a 95% confidence level to assess significant differences between treatments. To determine which treatments behaved differently and which ones yielded better results based on the study’s objective, Tukey’s multiple comparison test of means was performed.

## 3. Results and Discussion

### 3.1. Proximal Characterization

The proximal characterization of cassava bagasse (Table 3) indicates a moisture content of 87.28%. The dry matter is primarily composed of carbohydrates (70.28%) and fiber (25.72%), with low levels of proteins (1.88%) and fat (0.51%). These results are consistent with those reported by Refs. [20,21]. However, the estimated fiber content differs from that presented by Ref. [22] and is 20% higher than that reported by Polachini et al. [16]. These discrepancies may be attributed to factors such as the technology and efficiency of the raw material peeling and starch extraction processes used [23].

### 3.2. Drying Kinetics

The average equilibrium moisture content achieved by cassava bagasse subjected to drying from the different treatments was 10.64 ± 0.79%. The analysis of variance (ANOVA) revealed significant differences between the evaluated treatments regarding the drying time required to reach optimal moisture content (*p* < 0.05). The results of the Shapiro–Wilk and Bartlett tests confirmed that the errors met the assumptions of normality and homogeneity of variance, respectively. The results of Tukey’s test for the average drying times obtained by each treatment to reach equilibrium moisture content are specified in Table 4.

Tukey’s multiple comparison test of means indicated significant differences between treatment T0 and the treatments that used US or PEF prior to drying (T1, T2, T3, T4, T5, T6, T7, and T8). This suggests that applying US and PEF as pretreatments before convective drying significantly reduces the drying time of cassava bagasse. In particular, treatment T4, which utilizes 26 kHz probe US for 30 min, showed considerably shorter drying time compared to other treatments, with a 72.6% reduction compared to treatment T0 where cassava bagasse is dried without pretreatment (3.83 h versus 14.00 h).

**Figure 2:**
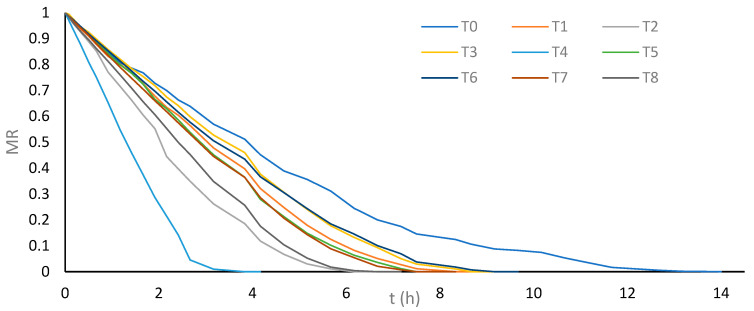
Average moisture loss ratio (MR) vs. drying time of cassava bagasse according to treatments.

In second place, in terms of shorter drying time, is treatment T2, using 37 kHz bath US for 30 min, with a 56% reduction compared to T0 (6.16 h versus 14.00 h). However, this treatment did not show a significant difference in drying time compared to treatment T8, which employs 7.5 kVcm^−1^ PEF for 30 min, achieving a 52.4% reduction compared to T0 (6.66 h versus 14.00 h). Figure 2 shows the drying kinetics of cassava bagasse for each evaluated treatment, highlighting the greater drop-in moisture loss rate (MR) for treatment T4, followed by treatments T2 and T8, respectively.

The drying time of 3.83 h achieved with treatment T4 is comparable to that obtained for cassava bagasse using combined filtration–pressing–drying technologies [24]. Additionally, the drying times of treatments T4 (3.83 h), T2 (6.16 h), and T8 (6.66 h) were shorter than the drying time of 6.9 h for cassava bagasse employing combined osmotic dehydration-drying technologies [20].

The results indicate that a 30 min US pretreatment is more effective for drying cassava bagasse than a 10 min treatment. Regarding the type of US equipment used, no significant differences were observed between bath US and probe US for a 10 min pretreatment, while for 30 min, 26 kHz probe US proved more effective than 37 kHz bath US. Regarding the emerging technology of PEF, the best results were obtained with a field intensity of 7.5 kVcm^−1^ for 30 min as a pretreatment.

### 3.3. Evaluation of Kinetic Models

Table 5 presents the parameters and statistical fit criteria of the kinetic models evaluated for the drying of cassava bagasse according to each treatment. The Wang and Singh model and the Page kinetic model were the most suitable for explaining moisture loss as a function of drying time, both with and without US and PEF pretreatments. These models showed the highest conformity for all treatments evaluated (R^2^ Adj > 0.99) and a low root mean square error (RMSE < 0.034).

The coefficients “b” and “k” in the Wang and Singh model and the Page model, respectively, indicate the drying rate of the product. Higher values of these coefficients correspond to a faster drying rate, which aligns with the shorter drying times observed in treatments T4, T2, and T8 that used US or PEF pretreatments.

The parameter “a” in the Wang and Singh model is another model fitting constant, useful for determining drying time. The lowest values of this constant were obtained in treatments T4, T2, and T8, corresponding to the treatments with the highest drying rates, while its highest value was for treatment T0, which did not use pretreatment and had the lowest drying rate. On the other hand, the parameter “n” in the Page model is related to the product’s structure and also influences drying time. The lower “n” values obtained in treatments with emerging US and PEF technologies indicate structural changes in the sample that promote greater mass transfer and faster drying.

These findings are consistent with the results reported by Oladejo et al. [25], who identified the Page model as the most suitable for drying cassava with ultrasound pretreatment, supporting the choice of this model to explain the results obtained in this study.

### 3.4. Effective Diffusivity

The ANOVA revealed significant differences between the evaluated treatments (*p* < 0.05) in terms of effective diffusivity (*Dv*). The average *Dv* for each treatment, organized according to the results of Tukey’s multiple comparison test, is shown in Table 6.

The results indicate that the treatment using 26 kHz probe US for 30 min (T4) exhibits a higher *Dv* (15.30 × 10^−10^ m^2^/s), resulting in more effective moisture displacement through the sample. Not only was the *Dv* of T4 higher, but it was also statistically different from the diffusivities obtained in the other treatments.

No significant differences were found between the *Dv* achieved by the treatment using 37 kHz bath US for 30 min (T2) and the treatment applying 7.5 kVcm^−1^ PEF for 30 min (T8). The *Dv* results confirm that treatments T4, T2, and T8 are the most effective in improving the efficiency of the cassava bagasse drying process by using US and PEF as pretreatments.

### 3.5. Energy Consumption

The energy consumption (E, kWh) determined for treatments T0, T4, T2, and T8 is illustrated in Table 7, where the energy saving percentage is established in relation to the energy consumption of treatment T0, in which the product is dried without any pretreatment.

The energy savings as an economic indicator of using US and PEF shows that pretreatment with a 26 kHz probe US for 30 min (T4) generates an energy savings of 30.46 kWh compared to treatment T0, which dries cassava bagasse without pretreatment 11.54 kWh vs. 42.00 kWh, respectively. Similarly, the pretreatment with 37 kHz bath US for 30 min (T2) results in an energy reduction of 55.9% compared to T0 18.52 kWh vs. 42.00 kWh, respectively, and with treatment T8, using 7.5 kVcm^−1^ PEF for 30 min, energy consumption is reduced by 52.4% 19.99 kWh vs. 42.00 kWh, respectively.

### 3.6. Evaluation of Functional Group Spectra

Fourier Transform Infrared (FTIR) has been recognized as a non-destructive mode of exploration that could deliver us both the quantitative and qualitative particulars about the subjected specimens. An infrared absorption spectrum has commonly been achieved by providing information about the presence of active sites or functional groups, chemical compositions, chemical structure, purity of compound, the number of targeted molecules, and overall molecular behavior [26].

**Figure 3:**
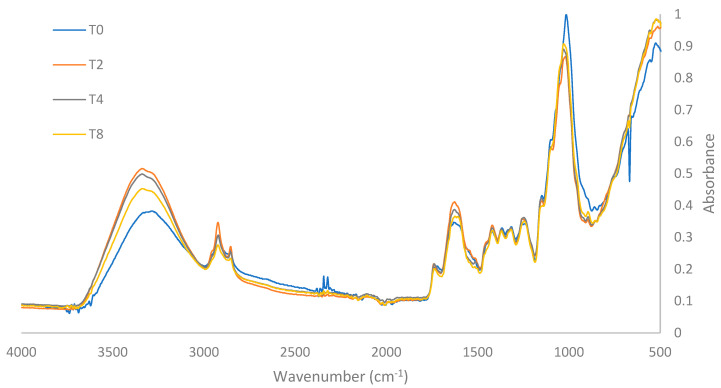
FTIR spectra of T0 and best treatments.

The infrared spectra (FTIR) of both treatment T0 and the most effective treatments using US and PEF (T2, T4, and T8) are illustrated in Figure 3. The effects of US and PEF on the functional groups present in the cassava bagasse samples are evidenced by changes in the characteristic frequency peaks of each functional group.

In the spectra acquired in the functional group frequency region between 4000 and 1500 cm^−1^, prominent peaks appear at 3300 cm^−1^, 2900 cm⁻^1^, 2850 cm^−1^, 1730 cm⁻^1^, and 1620 cm^−1^. The peak at 3300 cm^−1^ corresponds to the stretching of hydroxyl –OH groups present in the amylose and amylopectin of starch, as well as in the cellulose molecules of cassava bagasse [27]. The presence of hydroxyl groups has been observed to be closely linked with the cellulose molecules present in the raw fiber [28], which are part of the molecular structure of cassava bagasse [29]. Studies by Ref. [30] attribute this peak to the –OH stretching of glucopyranose rings in polysaccharides, including cellulose and starch.

Fronza [31] notes that the band between 3000 and 3500 cm^−1^ reveals the hydrophilic nature of starch and other components such as phenols, hemicellulose, cellulose, and lignin, indicating a higher fiber content as this band shifts. The “T0 Treatment” showed lower intensity at this peak compared to the other treatments.

Treatments with US and PEF demonstrated structural changes in the cassava bagasse fiber, decreasing starch content and increasing fiber proportion, which contributes to lower hydrophilicity and, consequently, greater drying efficiency. This also affects the fiber-to-starch ratio, with lower starch content and higher fiber proportion compared to the “T0 Treatment”. These findings align with studies by Edhirej at al. [32] and Travalini et al. [33], which associate the –OH stretching at 3300 cm^−1^ with higher cellulose concentration in the bagasse. However, they contrast with Carvalho et al. [34], who found a reduction in the intensity of this peak as fiber content decreases in the samples.

The peaks at 2900 and 2850 cm^−1^ represent the stretching of C–H bonds, and their intensity is related to the presence of the amorphous fraction of cellulose [35]. These are typical stretches of hemicellulose and cellulose [36] and can also be attributed to the stretching of methyl and methylene groups in lignocellulosic structures [27] or polysaccharides [37]. These peaks were similar, showing no significant changes between the spectra of treatments T0, T2, T4, and T8, indicating that the C–H molecular structure of hemicellulose and cellulose in cassava bagasse is not affected by treatments with 26 kHz probe US for 30 min (T4), 37 kHz bath US for 30 min (T2), or 7.5 kV cm^−1^ PEF for 30 min (T8), respectively.

The peak at 1730 cm^−1^ corresponds to the C=O bond characteristic of the molecular structure of lignin [33]. This band is attributed to the acetyl and uronic ester groups of hemicellulose and pectins or to the ester bond of the carboxylic group in ferulic and p-coumaric acids of lignin and/or hemicellulose [35,37]. The intensity of this peak was similar in treatments T0, T2, T4, and T8, indicating that the application of US and PEF under these conditions does not significantly affect the C=O molecular structure of hemicellulose and lignin present in the bagasse.

The presence and intensity of the 1620 cm^−1^ peak indicate the –OH deformation in the molecular structure of starch; the greater the intensity of this peak, the greater the respective deformation. This peak was highest in treatment T4, followed by treatments T2, T8, and T0. The higher intensity of this peak suggests greater –OH deformation for the starches [30] and a less hydrophilic nature [32,38], which partly explains the shorter drying times required when applying US and PEF pretreatments. This peak is also characteristic of the vibration of the aromatic rings in the lignin present in the samples [33,36]. In the spectra obtained in the fingerprint region between 1500 and 500 cm^−1^, four consecutive peaks of similar intensity (1422, 1370, 1318, and 1242 cm^−1^) and one highly intense peak at 1020 cm^−1^ are evident.

According to various FTIR studies conducted on cassava bagasse, the band corresponding to the 1422 cm^−1^ peak is characteristic of the presence of aromatic rings and C–H bonds typical of the molecular structure of lignin and cellulose, respectively [36]. The frequency corresponding to the 1318 cm^−1^ peak represents the C–H bending of cellulose [39], although the 1338 cm^−1^ peak, similar to the 1242 cm^−1^ peak, is characteristic of the syringyl and guaiacyl lignin structures, respectively [40]. The band corresponding to the 1242 cm^−1^ peak represents the C–O stretching vibration of hemicellulose components or the molecular structure of lignin [35,39]. Studies reported by Ref. [33] indicate that this peak indicates the presence of lignin in the sample and is indicative of vibrations and elongation of the C–O–C ether bonds.

Among treatments T0, T2, T4, and T8, no significant differences or changes were evident in the spectra of frequencies 1422, 1370, 1318, and 1242 cm^−1^. This indicates that the C–H and C–O molecular structure of lignin and hemicellulose present in the bagasse is not significantly affected by the US and PEF pretreatments used.

The prominent peak at 1020 cm^−1^ corresponds to the stretching of C–O and C–C bonds in the glucopyranose rings of amylose and amylopectin present in the molecular structure of cassava starch [30,33,34,38]. This peak decreases according to the degree of starch reduction in the sample [40]. The 1020 cm^−1^ peak was higher in treatment T0, indicating that the pretreatments with US (T2 and T4) and PEF (T8) affect the starch content of the bagasse, especially the US pretreatments T4 and T2, respectively.

### 3.7. Evaluation of Starch Content

The analysis of variance (ANOVA) indicated the presence of significant differences (*p* < 0.05) for the residual starch content results of the dried cassava bagasse samples obtained from treatments T0, T2, T4, and T8. The Tukey test results are illustrated in Table 8, and they indicate that all treatments were statistically different from each other for residual starch content.

The use of US and PEF pretreatments resulted in a significant decrease in residual starch content in the dried cassava bagasse samples. The results showed a greater starch loss when using US with a probe at 26 kHz for 30 min (T4) with a starch reduction percentage of 61.10% compared to the treatment T0 where no pretreatments were used (19.87 ± 0.70% vs. 51.08 ± 1.42%). Meanwhile, using a bath-type US at 37 kHz for 30 min (T2) resulted in a starch reduction percentage of 48.78% (26.16 ± 1.57% vs. 51.08 ± 1.42%). The lowest reduction in residual starch was obtained from the PEF treatment at 7.5 kVcm^−1^ for 30 min (T8), with a starch reduction percentage of 8.16% compared to treatment T0 (46.91 ± 0.85% vs. 51.08 ± 1.42%).

Refs. [40,41,42] reported that the application of US causes damage to the fibrous structure of the product, leading to the loss of residual starch due to pore rupture and formation of microscopic channels in the fibrous structure, resulting in the loss of cell adhesion due to the generation of large intercellular spaces. Similarly, Jeong et al. [43] state that PEF causes structural damage to the cell membrane, creating porosity in the tissue, and increasing mass transport channels, with an accelerated effect on mass transfer processes, thus facilitating the extraction of bio-compounds. This explains, in addition to the higher moisture loss, the starch loss in the bagasse samples previously treated with US and PEF.

The starch percentage obtained (51.08%) from dried cassava bagasse in “Treatment T0” is similar to the 50.77% reported by Srack et al. [40] but lower than the 60.68% obtained by Souto et al. [44] and higher than the 41.24% reported by Paternina-Contreras et al. [22]. These differences may stem from variations in efficiencies and processes of cassava starch extraction, as well as the techniques employed for its determination.

### 3.8. Morphological Characterization by Field Emission Scanning Electron Microscopy (FESEM)

**Figure 4:**
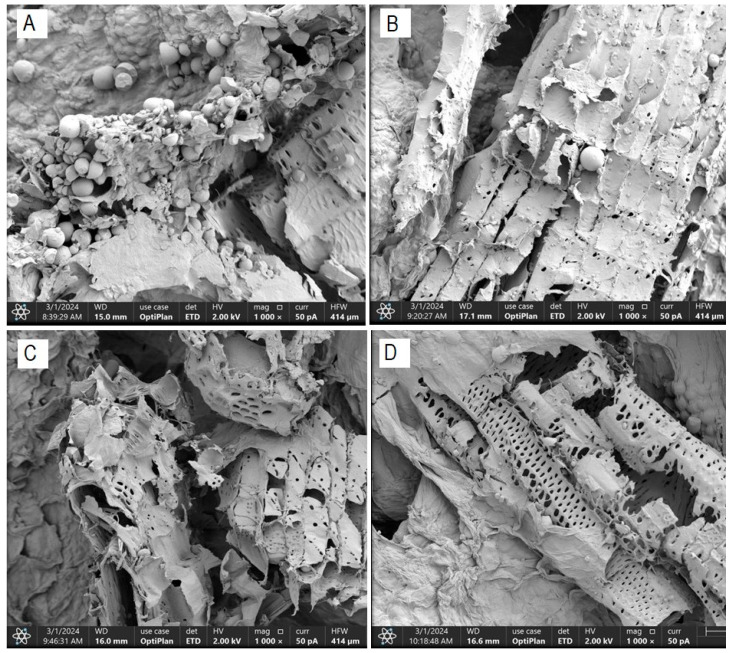
FESEM scanning electron micrograph—residual starch content ((**A**): T0; (**B**): T4; (**C**): T2 and (**D**): T8).

The morphological characterization conducted on dried cassava bagasse reveals the formation of a fibrous structure and a high presence of round and irregularly shaped starch granules, trapped within the bagasse fibers (Figure 4). These findings are consistent with those obtained by Weligama Thuppahige et al. [30] and Versino and García [37], who reported a significantly high amount of residual starch in the bagasse. However, several starch-free areas are also observed in the cell walls.

**Figure 5:**
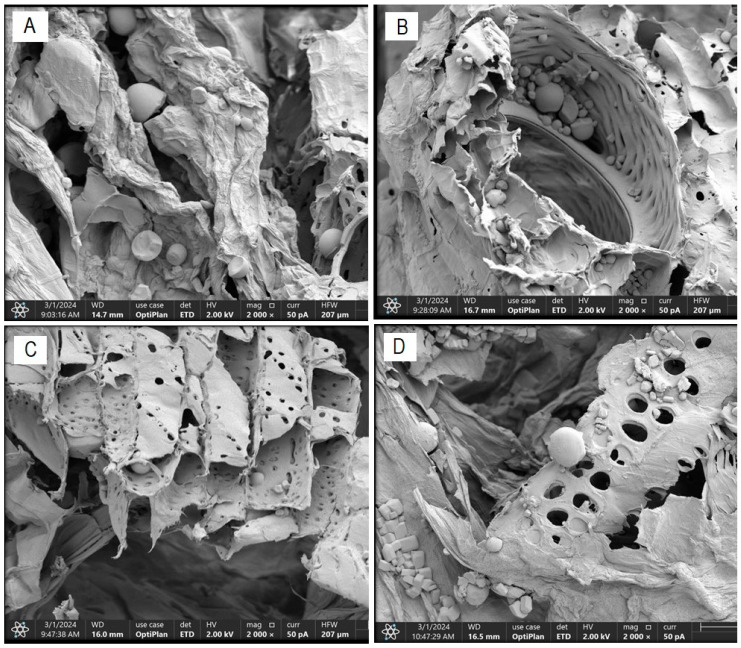
FESEM scanning electron micrograph—structural changes in cassava bagasse fiber ((**A**): T0; (**B**): T4; (**C**): T2 and (**D**): T8).

Figure 5 illustrates the alterations in the fibrous structure of cassava bagasse generated by the US and PEF pretreatments on said structure. The morphological characterization of the bagasse in “Treatment T0” (Figure 5A) indicates that this residue consists of an irregular fibrous structure with some equally irregular pores and certain cracks of an average size of 20.19 µm and starch granules of an average size of 17.89 µm, respectively. These results fall within the ranges reported by Versino and García [37] for cassava bagasse.

Figure 5B–D shows scanning electron micrographs of bagasse samples obtained from treatments with a probe-type US at 26 kHz for 30 min (T4), a bath-type US at 37 kHz for 30 min (T2), and PEF treatment at 7.5 kVcm^−1^ for 30 min (T8), respectively. The effect of the US treatment at 26 kHz for 30 min (T4) on the cellular structure of the cassava bagasse fiber is highlighted, which allows for greater efficiency in the diffusion and mass transfer processes that accompany its drying process. This explains the results of higher effective diffusivity, higher drying rate, and greater energy efficiency of this treatment compared to the others. Acoustic intensity causes an energy collapse in the cellular structure of the fibrous matrix of cassava bagasse, resulting in interfacial turbulence, disintegration of the outer material, energy dissipation, and increased diffusion and mass transfer [45].

## 4. Conclusions

The proximal characterization of cassava bagasse revealed a moisture content of 87.28%, with a predominant composition of carbohydrates (70.28%) and fiber (25.72%), along with low levels of proteins (1.88%) and fat (0.51%). Pretreatments with ultrasound (US) and pulsed electric fields (PEF) alter the molecular structure of the bagasse, causing disruptions in the fibrous structure of the bagasse, leading to the formation of microchannels and micropores, reducing material hydrophilicity, and thus improving mass transfer and consequently the efficiency of cassava bagasse drying, significantly increasing the drying rate and reducing drying time by up to 72% when using probe-type US at 26 kHz for 30 min. In this context, the Wang and Singh kinetic models and the Page model are suitable for describing the drying kinetics of cassava bagasse with and without US and PEF pretreatments. Additionally, a significant reduction in residual starch was observed, particularly notable with the application of probe-type ultrasound.

These results suggest that the use of US and PEF as pretreatments is an effective strategy to enhance the efficiency of cassava bagasse drying, which could have significant implications for the agri–food industry in terms of energy savings and process optimization.

Once the effectiveness of using US and PEF as pretreatments to enhance the efficiency of cassava bagasse drying has been demonstrated, future work can take several directions, such as the following:

- Investigate the optimization of specific parameters of ultrasound and pulsed electric field pretreatments aiming for increased efficiency in cassava bagasse drying. This would include varying the intensity, duration, and frequency of pretreatments to determine the optimal conditions.
- Explore other techniques as pretreatments specifically for cassava bagasse, such as microwave heating, UV irradiation, or enzymatic treatment, to further enhance drying efficiency.

## Figures and Tables

**Table 1:** Evaluated drying kinetics models.

Model	Equation
Newton	*MR* = *exp*(−*k t*)
Two-term exponential	*MR* = *exp*(-*kt*) + 1(1 − *a*) *exp*(−*kat*)
Wang and Singh	*MR =* 1 + *at + bt*^2^
Approximate Diffusion	*MR* = *a exp*(−*kt*) + 1(1 − *a*) *exp*(−*kbt*)
Page	*MR* = *exp*(−*kt^n^*)
Modified Page	*MR* = *exp*(−*kt*)*^n^*
Henderson and Pabis	*MR* = *a exp*(−*kt*)
Henderson and Modified Pabis	*MR* = *a exp*(−*kt*) + *b exp*(−*gt*) + *c exp*(−*ht*)
Logarithmic	*MR* = *a exp*(−*kt*) + *c*

*MR* is the moisture ratio; *t* is the drying time; and *k*, *a*, *b*, *c*, *g*, *h*, and *n* are model fitting constants.

**Table 2:** Description of treatments evaluated for the drying of cassava bagasse.

Treatment	Description
T0	Conventional drying of cassava bagasse without pretreatment.
T1	Pretreatment with bath-type US at 37 kHz for 10 min
T2	Pretreatment with bath-type US at 37 kHz for 30 min
T3	Pretreatment with probe-type US at 26 kHz for 10 min
T4	Pretreatment with probe-type US at 26 kHz for 30 min
T5	Pretreatment with PEF at 2.5 kVcm^−1^ for 10 min
T6	Pretreatment with PEF at 2.5 kVcm^−1^ for 30 min
T7	Pretreatment with PEF at 7.5 kVcm^−1^ for 10 min
T8	Pretreatment with PEF at 7.5 kVcm^−1^ for 30 min

**Table 3:** Average proximate characterization of cassava bagasse.

Component	Content (%) ± Standard Deviation
Humidity (wet basis)	87.28 ± 0.37
Dry matter (wet basis)	12.72 ± 0.38
Ash (dry basis)	1.62 ± 0.02
Protein (dry basis)	1.88 ± 0.25
Fat (dry basis)	0.51 ± 0.08
Fiber (dry basis)	25.72 ± 0.29
Carbohydrates (dry basis)	70.27 ± 0.34

**Table 4:** Average drying time of cassava bagasse according to each treatment.

Treatments	t (h) ± Standard Deviation
T0	14.00 ± 0.29 ^a^
T6	9.16 ± 0.50 ^b^
T3	8.66 ± 0.50 ^b^
T1	8.33 ± 0.29 ^bc^
T5	7.50 ± 0.29 ^cd^
T7	7.50 ± 0.29 ^cd^
T8	6.66 ± 0.50 ^de^
T2	6.16 ± 0.50 ^e^
T4	3.83 ± 0.29 ^f^

Means with different letters are significantly different (*p* < 0.05).

**Table 5:** Parameters and conformity criteria of kinetic models evaluated.

Model	Parameters	T0	T1	T2	T3	T4	T5	T6	T7	T8
Newton	k	0.2136	0.2724	0.3893	0.2472	0.6363	0.2829	0.2534	0.2887	0.3380
	R^2^adj	0.9820	0.9560	0.9649	0.9523	0.9571	0.9527	0.9636	0.9546	0.9538
	RMSE	0.0482	0.0759	0.0684	0.0793	0.0767	0.0790	0.0692	0.0776	0.0785
Two-term exponential	a	0.9515	0.9306	0.9257	0.9221	0.9236	0.9219	0.9291	0.9246	0.9249
	k	0.2259	0.2965	0.4260	0.2720	0.6949	0.3115	0.2762	0.3166	0.3704
	R^2^adj	0.9850	0.9629	0.9726	0.9615	0.9641	0.9618	0.9710	0.9628	0.9617
	RMSE	0.0440	0.0697	0.0604	0.0712	0.0702	0.0711	0.0619	0.0702	0.0716
Wang and Singh	a	−0.1569	−0.1973	−0.292	−0.176	−0.4739	−0.2045	−0.1848	−0.2091	−0.2471
	b	0.0062	0.0088	0.0207	0.0067	0.0541	0.0093	0.0080	0.0099	0.0140
	R^2^adj	0.9984	0.9953	0.9957	0.9950	0.9919	0.9944	0.9975	0.9951	0.9943
	RMSE	0.0142	0.0248	0.0285	0.0257	0.0334	0.0273	0.0181	0.0254	0.0276
Approximate diffusion	a	14.8500	10.5000	7.7480	69.8600	7.9970	22.9000	16.8800	14.0200	10.3100
	b	0.9438	0.8365	0.8391	0.9713	0.8419	0.9201	0.9098	0.8753	0.8424
	k	0.0999	0.0794	0.1478	0.0608	0.2383	0.0766	0.0788	0.0820	0.1036
	R^2^adj	0.9963	0.9929	0.9916	0.9932	0.9850	0.9919	0.9951	0.9926	0.9911
	RMSE	0.0220	0.0305	0.0334	0.0300	0.0454	0.0328	0.0253	0.0314	0.0344
Page	k	0.1347	0.1450	0.2549	0.1207	0.4931	0.1469	0.1388	0.1558	0.1935
	n	1.2850	1.4920	1.4650	1.5220	1.5290	1.5350	1.4390	1.5080	1.5300
	R^2^adj	0.9964	0.9925	0.9967	0.9929	0.9932	0.9945	0.9948	0.9931	0.9934
	RMSE	0.0216	0.0313	0.0209	0.0307	0.0305	0.0271	0.0261	0.0303	0.0296
Modified Page	k	0.3676	0.3683	0.7945	0.3900	0.8546	0.2016	0.1711	0.4010	0.2635
	n	0.5814	0.7394	0.4900	0.6338	0.7446	1.4030	1.4810	0.7200	1.2830
	R^2^adj	0.9815	0.9541	0.9631	0.9504	0.9543	0.9507	0.9622	0.9526	0.9516
	RMSE	0.0489	0.0775	0.0700	0.0808	0.0792	0.0807	0.0706	0.0792	0.0804
Henderson and Pabis	a	1.0490	1.0700	1.0750	1.0780	1.0770	1.0790	1.0710	1.0760	1.0750
	k	0.2254	0.2953	0.4238	0.2706	0.6912	0.3098	0.2749	0.3150	0.3684
	R^2^adj	0.9854	0.9629	0.9726	0.9615	0.9641	0.9618	0.9710	0.9629	0.9617
	RMSE	0.0440	0.0700	0.0604	0.0712	0.0702	0.0710	0.0618	0.0701	0.0715
Modified Henderson and Pabis	a	−17.420	−0.133	−0.993	−25.080	−8.099	−9.585	−1.938	−0.434	−8.177
	b	−0.1766	−4.8040	12.720	26.520	9.289	10.5	−0.1459	−1.253	−0.3262
	c	18.5800	5.924	−10.71	−0.4168	−0.180	0.1593	3.1110	2.6850	9.4940
	g	1.3890	0.1033	0.2688	0.3081	0.3402	0.1314	−0.0631	0.4905	1.0030
	h	0.2374	0.1301	0.2417	0.8358	1.9790	0.4612	0.1829	0.2934	0.1998
	k	0.2367	1.4600	0.5648	0.3068	0.3012	0.1202	0.1864	0.0597	0.1762
	R^2^adj	0.9910	0.9956	0.9961	0.9782	0.9904	0.9809	0.9941	0.9969	0.9964
	RMSE	0.0342	0.0240	0.0229	0.0536	0.0363	0.0503	0.0279	0.0201	0.0218
Logarithmic	a	1.171	1.531	1.323	1.636	1.338	1.561	1.424	1.53	1.488
	c	−0.1547	−0.511	−0.287	−0.6113	−0.2977	−0.5332	−0.3994	−0.5041	−0.460
	k	0.1627	0.1421	0.2619	0.1194	0.423	0.1473	0.1472	0.1533	0.1877
	R^2^adj	0.9959	0.993	0.9929	0.9939	0.9864	0.9928	0.9956	0.9932	0.9918
	RMSE	0.0232	0.0302	0.0307	0.0283	0.0432	0.0309	0.0241	0.0300	0.0331

**Table 6:** Tukey’s mean comparison test for *Dv*.

Treatments	*Dv* (m^2^/s)X10^10^ ± Standard Deviation
T4	15.30 ± 2.41 ^a^
T2	9.39 ± 1.47 ^b^
T8	8.16 ± 1.28 ^bc^
T7	6.97 ± 1.09 ^cd^
T5	6.85 ± 1.08 ^cd^
T1	6.56 ± 1.02 ^cd^
T6	6.10 ± 0.95 ^d^
T3	5.98 ± 0.94 ^d^
T0	5.08 ± 0.74 ^d^

Means with different letters are significantly different (*p* < 0.05).

**Table 7:** Energy consumption.

Treatment	Energy Consumption (*E*, kWh)			Energy Saving (%)
	Pretreatment	Drying	kWh Total	
T4	0.05	11.49	11.54	72.5
T2	0.04	18.48	18.52	55.9
T8	0.01	19.98	19.99	52.4
T0	—	42.00	42.00	—

**Table 8:** Tukey’s mean comparison test for starch content.

Treatments	Starch (%) ± Standard Deviation
T0	51.08 ± 1.42 ^a^
T8	46.91 ± 0.85 ^b^
T2	26.16 ± 1.57 ^c^
T4	19.87 ± 0.70 ^d^

Means with different letters are significantly different (*p* < 0.05).

## Data Availability

The original contributions presented in the study are included in the article, further inquiries can be directed to the corresponding author.
